# Predicting total lung capacity from spirometry: a machine learning approach

**DOI:** 10.3389/fmed.2023.1174631

**Published:** 2023-05-19

**Authors:** Luka Beverin, Marko Topalovic, Armin Halilovic, Paul Desbordes, Wim Janssens, Maarten De Vos

**Affiliations:** ^1^Statistics Research Centre, KU Leuven, Leuven, Belgium; ^2^ArtiQ NV, Leuven, Belgium; ^3^Laboratory of Respiratory Diseases and Thoracic Surgery, Department of Chronic Diseases Metabolism and Ageing, Ku Leuven, Leuven, Belgium; ^4^Stadius, Department of Electrical Engineering, KU Leuven, Leuven, Belgium; ^5^Department of Development and Regeneration, KU Leuven, Leuven, Belgium

**Keywords:** restriction, spirometry, machine learning, interstitial lung disease, total lung capacity

## Abstract

**Background and objective:**

Spirometry patterns can suggest that a patient has a restrictive ventilatory impairment; however, lung volume measurements such as total lung capacity (TLC) are required to confirm the diagnosis. The aim of the study was to train a supervised machine learning model that can accurately estimate TLC values from spirometry and subsequently identify which patients would most benefit from undergoing a complete pulmonary function test.

**Methods:**

We trained three tree-based machine learning models on 51,761 spirometry data points with corresponding TLC measurements. We then compared model performance using an independent test set consisting of 1,402 patients. The best-performing model was used to retrospectively identify restrictive ventilatory impairment in the same test set. The algorithm was compared against different spirometry patterns commonly used to predict restriction.

**Results:**

The prevalence of restrictive ventilatory impairment in the test set is 16.7% (234/1402). CatBoost was the best-performing machine learning model. It predicted TLC with a mean squared error (MSE) of 560.1 mL. The sensitivity, specificity, and F1-score of the optimal algorithm for predicting restrictive ventilatory impairment was 83, 92, and 75%, respectively.

**Conclusion:**

A machine learning model trained on spirometry data can estimate TLC to a high degree of accuracy. This approach could be used to develop future smart home-based spirometry solutions, which could aid decision making and self-monitoring in patients with restrictive lung diseases.

## 1. Introduction

Restrictive lung disorders are a group of conditions that affect the ability of the lungs to expand fully, resulting in reduced lung capacity and difficulty breathing. These conditions are typically caused by either intrinsic or extrinsic factors, such as interstitial lung diseases or chestwall problems (1). Patients with restrictive lung disorders often experience a decreased quality of life and increased morbidity, as the reduced lung capacity can make it difficult for them to engage in physical activities and perform everyday tasks (2). While the true population prevalence of restrictive diseases is unknown, it is estimated that the occurrence is 3–6 persons per 100,000 in the United States (3).

The diagnostic criterion for restrictive lung disease is a total lung capacity (TLC) that falls below the lower limit of normal (LLN), which is defined as the fifth percentile of a healthy population. The measurement of TLC can be obtained using five different standardized methods: whole-body plethysmography (WBP), helium dilution, nitrogen gas washout, chest radiographs, and computed tomography scanning (4, 5). However, these methods are not widely available in primary care, require expert knowledge and are costly for routine use. As a result, primary care clinicians often rely on spirometry results to identify potential cases of lung restriction and decide which patients should undergo further pulmonary function testing.

In recent years, the use of home-based spirometry to monitor lung function in patients with interstitial lung disease (ILD) has gained attention in clinical practice and research (6–8). Home-based spirometry has the potential to increase convenience and accessibility for patients with ILD, improve the frequency of data collection, and make it easier for patients to receive regular assessments of their lung function. In addition, the integration of smartphone applications has facilitated communication and collaboration between patients and healthcare providers. With advances in machine learning (ML) and an increasing amount of health data available for analysis, it is becoming more feasible to use ML algorithms to improve both the quality and the interpretation of pulmonary function testing (9, 10). Despite the potential benefits of using ML in home-based spirometry, most research has focused on automating current human tasks (e.g., diagnosis). Besides, ML approaches have also the potential to estimate non-standard values that have clinical impact, like TLC values.

The objective of this study was to train a supervised ML model to predict TLC values using patient characteristics and data from spirometry. The secondary objective was to investigate whether these predictions could be used to accurately identify restrictive lung impairment defined as TLC < LLN, where reference values are derived from the 2012 global lung initiative (GLI) equations (11). We evaluate the performance of our model on an independent dataset and compare its ability to identify restrictive lung impairment to commonly used clinical guidelines (2005 ATS/ERS standards). Overall, our study investigates the potential use of ML to aid in decision-making in office and home-based spirometry by providing accurate and timely predictions of TLC. Moreover, it allows to investigate in which patient population such ML-based prediction might be most beneficial.

## 2. Methods

### 2.1. Data collection and preprocessing

In this study, we obtained data from two different sources: ArtiQ^1^ and University Hospital Leuven. The data from ArtiQ is used to train and tune the ML models, whereas the Hospital data is used as an independent test set to evaluate each model’s ability to predict TLC and subsequently identify restrictive lung impairment. Both datasets contain only Caucasian patients. The training data collected from ArtiQ consists of patient characteristics and spirometry measurements with a known TLC value. To detect anomalies, we implemented the Isolation Forest algorithm with 100 base estimators (12). We removed all observations with an anomaly score at or above the 99th percentile. After pre-processing, we were left with 51,761 unique observations where each observation represented a different patient.

The independent test data set consists of 1,402 patients who performed spirometry and WBP. This data set is formed by combining two different cohorts that were studied in previous work:

1. a prospective cohort study on first-time admissions in a population-based sample (13), and
2. a retrospective cohort study of PFT data (14).

More details on the studies can be found in the corresponding publications. Each subject had a validated clinical diagnosis based on their medical history and complete PFT. Collected data for testing the models are from studies approved by the Ethics Committee of the University Hospital in Leuven. The combined cohort data set included patients diagnosed with obstructive (*n* = 885) and restrictive (*n* = 288) lung disorders, as well as healthy individuals (*n* = 229). All patients included in the studies provided informed consent. A cohort description is provided in Table 1.

**Table 1:** Data are presented as mean +/-standard deviation, or number (%).

Characteristics	1,402 subjects present in the cohort		1,108 subjects with no restrictive lung impairment		234 subjects with restrictive lung impairment	
Sex	Male: 820 (58%)	Male: 586 (50%)	Male: 147 (63%)
	Female: 582 (42%)	Female: 586 (50%)	Female: 87 (37%)
Age (y)	54.8 ± 16.0	54.5 ± 16.3	56.2 ± 14.7
Height (cm)	168.8 ± 9.6	168.6 ± 9.7	170 ± 9.3
BMI (kg/m^2^)	26.38 ± 5.31	26.11 ± 5.25	27.73 ± 5.58
FVC (L)	3.41 ± 1.09	3.57 ± 1.15	2.62 ± 0.79
FEV₁ (L)	2.45 ± 0.95	2.53 ± 1.02	2.05 ± 0.62
FEV₁/FVC	71.39 ± 13.20	69.86 ± 13.99	79.02 ± 9.24
TLC (L)	5.86 ± 1.36	6.20 ± 1.44	4.15 ± 0.96

Baseline characteristics and lung function measurements in patients with and without restrictive ventilatory impairment are defined by total lung capacity less than lower limit of normal (TLC < LLN). BMI, body mass index; FVC, forced vital capacity; FEV₁, forced expiratory volume in 1 s; TLC, total lung capacity.

### 2.2. Machine learning model training for TLC prediction

For the predicton of TLC, we trained three tree-based ML models - Random Forest (RF) (15), Extreme Gradient Boosting (XGBoost) (16), and Categorical Boosting (CatBoost) (17). These algorithms are well suited for tabular data sets, and are commonly used in industry, research, and competitions. The final feature set used for model training consisted of patient characteristics (e.g., age, height, gender, and weight) and well-known spirometry measurements (e.g., FVC, FEV₁, FEV₁/FVC, peak expiratory flow and forced expiratory flow at different percentages of FVC). For the XGBoost and RF models, one-hot encoding was applied to the categorical feature (gender). These were all the features available for use in the model training.

**Figure 1:**
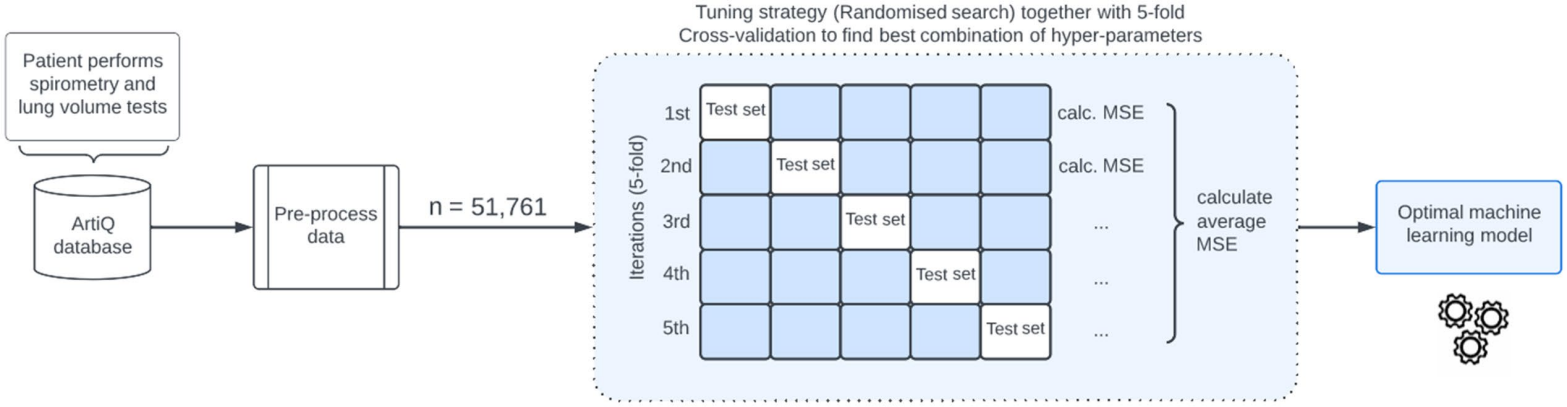
Illustration of the machine learning-based algorithm for predicting total lung capacity. MSE, mean squared error.

Hyper-parameters of the models were fine-tuned through a randomized search (18) with 220 sampled hyper-parameters. To develop the XGBoost model, a total of 43,200 possible combinations were considered. For the CatBoost and RF models, 30,870 and 672 possible combinations were considered, respectively. To find the optimal combination of hyper-parameters, we applied k-Fold Cross-Validation (k-fold CV) to the training data set (19). The value of k was set to 5 when performing k-fold CV because we found it to provide a good balance between computing time, bias, and variance. We then selected the hyper-parameters that resulted in the lowest CV mean squared error (MSE). The modeling process is depicted in Figure 1. For all models, we constructed hyper-parameter grid values that are in accordance with existing literature and best practices from competitive data science platforms such as Kaggle.^2^

### 2.3. Statistical analysis

Model development and statistical analyses were performed using the Python programming language. The MSE was used as a statistical measure for assessing the accuracy of our TLC prediction models. Low MSE values express a good fit of the model. To demonstrate the relationship between reference TLC values and the model predictions, the Pearson correlation coefficient was calculated. A value closer to 1 indicates better model performance.

In this study, the ground truth for restrictive lung impairment was defined as TLC < LLN (5th percentile), where the LLN for each patient was derived from the GLI reference values (11).

Two different definitions of restrictive patterns were compared to this ground truth. The first one is based on our model and is defined as TLC_predicted_ < LLN. The second one is based on the 2005 ERS/ATS standards [5], commonly used by physicians to identify patients, and is defined as FVC < LLN and FEV₁/FVC ≥ LLN.

The two proposed definitions were compared to the ground truth according to the confusion matrix as depicted in Table 2. For instance, if the predicted TLC values are below the LLN, this suggests that the ML model accurately identifies patients with restrictive lung impairment. From this confusion matrix, performance indicators can be calculated such as sensitivity, specificity, positive predictive value (PPV) and F1-score.

**Table 2:** Confusion matrix for the prediction of restriction.

	Prediction of restriction (TLC_predicted_ < LLN)	Prediction of no restriction (TLC_predicted_ ≥ LLN)
True restriction (TLC < LLN)	True positive	False negative
No true restriction (TLC ≥ LLN)	False positive	True negative

In this study, the ground truth for the prediction of a restriction is TLC < LLN.

## 3. Results

### 3.1. Prediction of the TLC using machine learning

The optimal hyperparameter configurations for the CatBoost and XGBoost models shared some similarities. For example, both models found 1,000 trees (or estimators) to be ideal, and the maximum depth of the tree in both configurations was 10. More details are given in Supplementary material. After fine-tuning, none of the models revealed any signs of overfitting, suggesting a satisfactory balance between bias and variance.

The three studied ML algorithms are assessed using the MSE. Among all models, CatBoost yielded the lowest MSE (MSE_CatBoost_ = 560.1, MSE_XGBoost_ = 569.6 and MSE_RF_ = 575.1). Therefore, we proceeded with this model for TLC predictions (TLC_CatBoost_).

**Figure 2:**
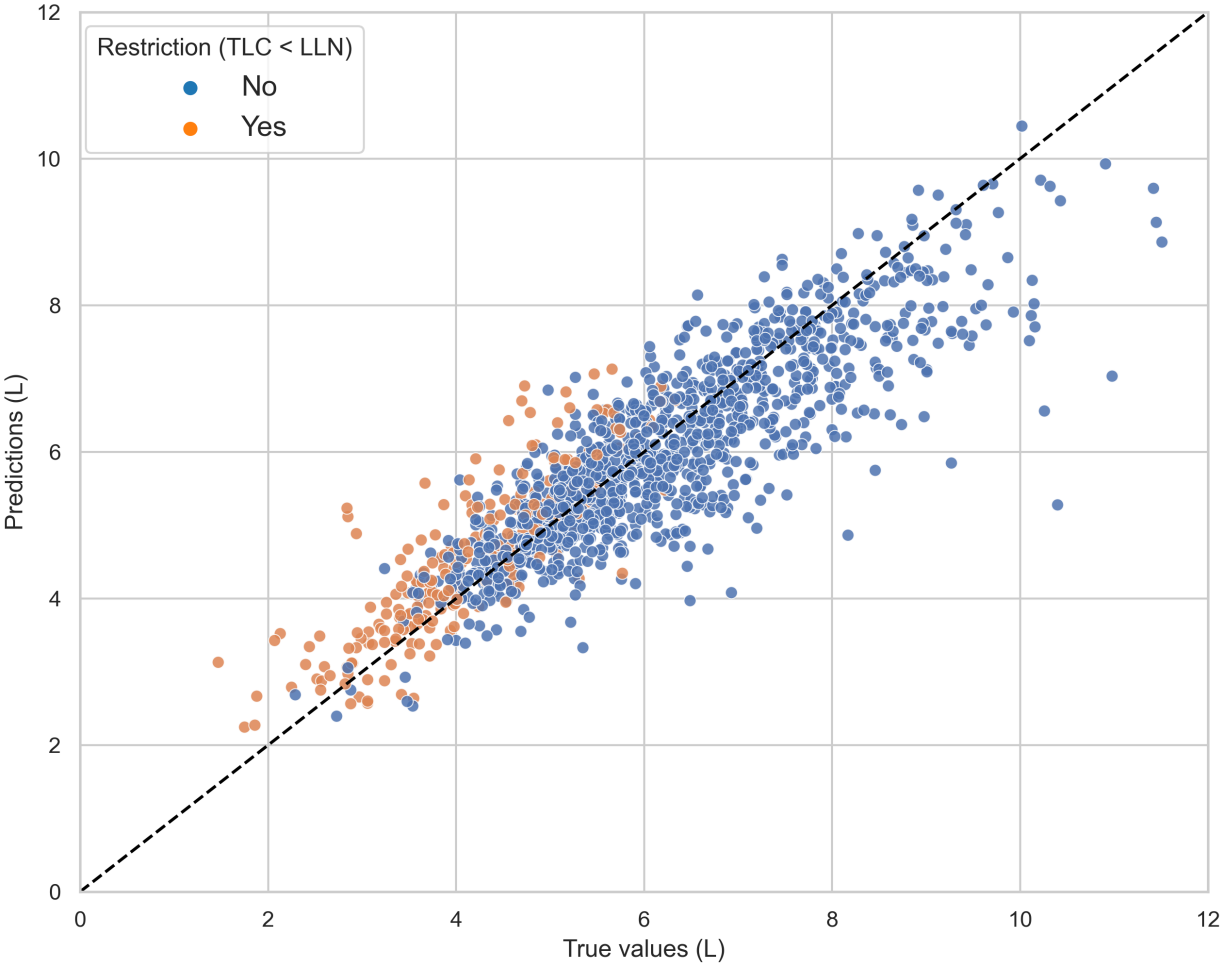
The total lung capacity (TLC) predictions of the CatBoost model (TLC_CatBoost_) against the reference TLC measurements in the independent test set, grouped by true restriction defined as TLC < lower limit of normal (LLN). The black dashed line represents the line of ideal agreement.

Model predictions and reference TLC values (range: 1.47–11.51 l) were highly correlated with a Pearson correlation coefficient of 0.88 (see Figure 2). The average difference between TLC_CatBoost_ and true TLC values was 107.2 mL.

**Figure 3:**
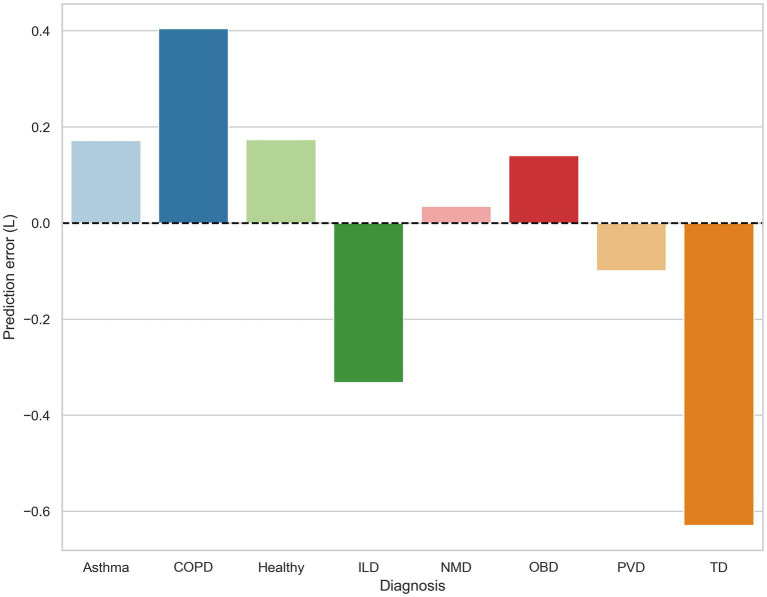
The prediction error for each diagnosis is calculated as the difference between the average total lung capacity (TLC) value and the average TLC_CatBoost_ prediction for that group. Bars above and below the horizontal dotted line indicate model underestimation and overestimation, respectively. COPD, chronic obstructive pulmonary disease; ILD, interstitial lung disease; OBD, other obstructive disease; NMD, neuromuscular disease; PVD, pulmonary vascular disease; TD, thoracic deformity.

Figure 3 shows that the magnitude of underestimation was highest in patients diagnosed with obstructive ventilatory impairments such as chronic obstructive pulmonary disorder (COPD) and asthma. In contrast, the model on average largely overestimated the TLC values for patients with restrictive disorders, including ILD and thoracic deformity.

### 3.2. Identifying restrictive ventilatory impairment

Confusion matrices for the different definitions are shown in Table 3. 16.7% (234/1402) of the 1,402 patients were detected as having restriction defined as TLC < LLN (5th percentile) compared to 13.8% (194/1402) with our algorithm and 18.0% (252/1402). Following the 2005 standards, 93 unnecessary full PFT would have been performed (PPV of 62%) versus only 35 with our method (PPV of 82%). Most of unnecessary tests would be done in patients diagnosed with asthma (32 patients) and COPD (20 patients). Those subjects will have a small airway obstructive syndrome or non-specific pattern, as previously described (20, 21).

**Table 3:** Confusion matrix for the prediction of restriction (a) based on our machine learning model and (b) based on the 2005 standards definition.

	Prediction of restriction (TLC_predicted_ < LLN)	Prediction of no restriction (TLC_predicted_ ≥ LLN)	Total
True restriction (TLC < LLN)	159	75	234
No true restriction (TLC ≥ LLN)	35	1,133	1,168
Total	194	1,208	1,402

	Prediction of restriction (FVC < LLN and FEV₁/FVC ≥ LLN)	Prediction of no restriction (FVC < LLN and FEV₁/FVC ≥ LLN)	Total
True restriction (TLC < LLN)	159	75	234
No true restriction (TLC ≥ LLN)	93	1,075	1,168
Total	252	1,150	1,402

Table 4 details the performance indicators (sensitivity, specificity, PPV and F1-score) for the studied approaches. Our baseline algorithm achieved the same sensitivity (68%) as the 2005 ERS/ATS guidelines for predicting restriction. However, our algorithm had higher specificity and attained a relatively good balance between sensitivity and PPV (F1-score of 74%). Moreover, lowering TLC estimations by subtracting *α* = 0.3 substantially increased the sensitivity of our algorithm from 68 to 83%. The algorithm’s ability to effectively rule out restriction was then only moderately reduced (specificity 92%).

**Table 4:** Overall performance of different definitions to identify restrictive ventilatory impairment defined by TLC < LLN.

Definition	Sensitivity (%)	Specificity (%)	PPV (%)	F1-score (%)
FVC < LLN and FEV₁/FVC ≥ LLN	68	93	63	65
TLC_CatBoost_ < LLN	68	97	82	74	TLC_CatBoost_ − α < LLN	83	92	67	75

The number of patients that would have missed necessary lung volume tests to confirm restriction when using the different definitions is shown in Table 5. Across all definitions, patients diagnosed with ILD were the most susceptible to false negative results. Of the 165 patients with ILD, the 2005 ERS/ATS guideline definition missed pulmonary restriction in 33 patients. Our baseline algorithm yielded a similar result; however, when α was adjusted to 0.3 the number of false negatives for ILD patients decreased almost threefold.

**Table 5:** Number of patients missed with restriction (TLC < LLN) in test data, grouped by disease subtype.

Disease diagnosis	FVC < LLN and FEV₁/FVC ≥ LLN	TLC_CatBoost_ < LLN	TLC_CatBoost_ − 0.3 < LLN
ILD	33 (44.6%)	32 (42.1%)	13 (33.3%)
TD	10 (13.5%)	7 (9.2%)	6 (15.4%)
NMD	1 (1.6%)	2 (2.6%)	–
Asthma	9 (12.2%)	14 (19.7%)	7 (17.9%)
Healthy	5 (6.8%)	5 (6.6%)	3 (7.7%)
COPD	10 (13.5%)	10 (13.2%)	8 (20.5%)
OBD	4 (5.4%)	3 (3.9%)	1 (2.6%)
PVD	2 (2.7%)	2 (2.6%)	1 (2.6%)
Total	74	76	39

COPD, chronic obstructive pulmonary disease; ILD, interstitial lung disease; OBD, other obstructive disease; NMD, neuromuscular disease; PVD, pulmonary vascular disease; TD, thoracic deformity; TLC, total lung capacity; LLN, lower limit of normal; FVC, forced vital capacity; FEV₁, forced expiratory volume in 1 s; TLC, total lung capacity; TLCCatBoost, CatBoost predicted total lung capacity.

## 4. Discussion

To the best of our knowledge, this is the first time that spirometry data has been investigated to estimate TLC values using ML models and large data sets. Given the type of data, our findings indicate that tree-based algorithms in general are well suited for the prediction task at hand. After evaluating the models using MSE, we found that the CatBoost model performed the best.

For patients diagnosed with pulmonary vascular disease and neuromuscular disease, the mean absolute difference between TLC values obtained by CatBoost and volume measurements was the lowest with 392.2 and 324.1 mL, respectively. However, in patients with COPD our TLC prediction model largely underestimated true TLC values. This finding might be explained by a phenomenon called pseudorestriction (22). Patients with severe obstruction may have air trapping with high residual volumes, thereby reducing FVC for a given increased TLC (4). To date, 228 patients were identified with low FVC (LLN) despite normal TLC, of which 49.6% had the diagnosis of COPD. These subjects contributed most to the underestimation we observe in the upper end of Figure 2.

Considering the satisfactory performance of our TLC prediction model, we examined its ability to serve as a tool for identifying restrictive lung impairment. We incorporated a linear correction term α to account for the model’s tendency to overestimate and underestimate in patients with and without restriction, respectively. By subtracting a small value of α to lower TLC predictions, the algorithm was able to achieve a remarkably high sensitivity without negatively impacting specificity; thereby transforming spirometry into a high-value screening test. The tuning constant α that controls the trade-off between sensitivity and specificity can be adjusted according to the context and priorities of different testing laboratories. For instance, an algorithm that emphasizes a higher specificity over sensitivity might be more desirable in rural or sparsely populated areas, where avoiding unnecessary referrals is important. In other scenarios, where PFTs are more developed and accessible, the algorithm can be tuned to prioritize sensitivity.

By using our algorithm to predict TLC from spirometry data, primary care providers can quickly and accurately identify patients who are likely to have a restrictive lung disease. For example, if a patient’s predicted TLC falls below a certain threshold, the ML algorithm could alert the patient and their healthcare provider, indicating that the patient should see a doctor for further evaluation and potential treatment. This can help to prevent diagnostic delays and ensure that patients receive timely and appropriate care. At the moment, diagnosing restrictive lung diseases is challenging, and many patients with ILD have experienced misdiagnosis, delayed treatment, and unnecessary tests on their path to a final diagnosis (23, 24). This methodology might be particularly useful to identify ILD. It is different from clinical standards that it directly estimates TLC, regardless of the preset spirometry criteria (FVC < LLN and FEV1/FVC > LLN) and will therefore also identify real restriction (proven TLC < LLN) in patients that are not having the preset spirometry disturbances. To document the difference, we checked in our test set in the group of patients with no restrictive spirometry (FVC > LLN) how many were still determined by the algorithm to have a TLC < LLN: 14 subjects. These 14 subjects would normally not have been referred for volumes. Interestingly, the majority (*n* = 11) of these patients had ILD. When we increase sensitivity of TLC CATboost with −0.3 alpha correction to 83% (by reducing the number of FN) compared to the ERS spirometry criteria, even a larger group of ILD patients can be identified.

Although our approach benefited from a large and varied data set for model building and parameter tuning, this study has some important limitations. First, our ML models were trained and tested mostly on Caucasian patients from a Belgium population. Therefore, the model’s ability to equally perform on other populations cannot be guaranteed. Second, the majority of the TLC measurements in the training data were obtained by whole body plethysmography. Although this method is often considered the gold standard, it has been shown to overestimate TLC in patients with obstructive diseases (25, 26). Moreover, we estimate that in the training dataset 20% of the volume measurements were obtained with Helium dilution technique, which might be less accurate. We expect this influence to be small, but cannot exclude that it results in an underestimation of volumes, particularly with obstructive airways disease. Hence, we do observe that volumes for obstructive airways diseases are underestimated when evaluated in the test set of which all data are plethysmography volumes. In our view it is less likely to play a role in restrictive diseases as it is know that discrepancies between plethysmography and volumes are less pronounced. Third, our volume data were obtained from the clinical routine of expert centers according to ERS/ATS standards, but no additional quality control was performed on the individual maneuvers which may have introduced some bias. Finally, we only investigated common ML algorithms and structured tabular data for developing our TLC prediction model. It is worth exploring the integration of unstructured data such as full flow-volume curves in combination with other prediction techniques like deep neural networks.

In conclusion, we have demonstrated that ML has the potential to estimate TLC from spirometry data and patient characteristics with high accuracy. Additionally, we showed that the TLC predictions can be used to identify restrictive ventilatory impairment with higher sensitivity and specificity than commonly used RSPs. Our solution can be integrated into smart spirometry software that is used at the level of the practicing physicians and home use spirometry. While adoption of such a tool may enable earlier diagnosis of restriction, further research studies are required to evaluate the accuracy and effectiveness of our model in predicting TLC and identifying restrictive lung impairment. This will help to determine whether the model can improve diagnostic accuracy and patient outcomes, and guide future research and development in this area.

## Data availability statement

The data analyzed in this study is subject to the following licenses/restrictions: patient confidentiality and participant privacy. Requests to access these datasets should be directed to ArtiQ, info@artiq.eu.

## Ethics statement

The studies involving human participants were reviewed and approved by www.clinicaltrials.gov. The patients/participants provided their written informed consent to participate in this study.

## Author contributions

LB: conceptualization, formal analysis, investigation, and writing original draft. MT and AH: data curation, methodology, resources and validation. PD and WJ: writing, reviewing, and editing. MV: supervision and editing. All authors contributed to the article and approved the submitted version.

## Conflict of interest

MT, AH, and PD were employed by the ArtiQ NV. MV has received consultancy fees from ArtiQ NV. WJ was a shareholder at ArtiQ NV.

The remaining author declares that the research was conducted in the absence of any commercial or financial relationships that could be construed as a potential conflict of interest.

## Publisher’s note

All claims expressed in this article are solely those of the authors and do not necessarily represent those of their affiliated organizations, or those of the publisher, the editors and the reviewers. Any product that may be evaluated in this article, or claim that may be made by its manufacturer, is not guaranteed or endorsed by the publisher.

## Footnotes

^1^ https://www.artiq.eu/

^2^ https://www.kaggle.com/
