# Supplementary material for: Predicting total lung capacity from spirometry: a machine learning approach
Source: Front Med (Lausanne). 2023 May 19;10:1174631. doi: 10.3389/fmed.2023.1174631 (PMC10238228; doi:10.3389/fmed.2023.1174631)
Supplement: Supplementary file 1 [file Data_Sheet_1.docx]

***Supplementary Material***

Predicting Total Lung Capacity from Spirometry: A Machine Learning Approach

**Luka Beverin¹, Marko Topalovic², Armin Halilovic², Paul Desbordes², Wim Janssens³, and Maarten De Vos*⁴**

*** Correspondence:** Corresponding Author: maarten.devos@kuleuven.be

# Supplementary Figures and Tables

**Hyper-parameter configurations**

Table S1. XGBoost model

| Hyper-parameters | Value ranges | Optimal value |
| --- | --- | --- |
| n_estimators | [200,500,1000] | 1000 |
| learning_rate | [0.01,0.02,0.03,0.1,0.3,0.6,1] | 0.01 |
| min_child_weight | [1,3,5,10,15] | 15 |
| gamma | [0,0.01,0.1,0.2] | 0.01 |
| subsample | [0.6,0.7,0.8,0.9,1,1.1] | 0.7 |
| colsample_by_tree | [0.6,0.7,0.8,0.9,1] | 0.9 |
| max_depth | [2,3,4,6,7,10] | 10 |

Table S2. CatBoost model

| Hyper-parameters | Value ranges | Optimal value |
| --- | --- | --- |
| n_estimators | [200,500,1000] | 1000 |
| learning_rate | [0.01,0.02,0.03,0.1,0.3,0.6,1] | 0.01 |
| min_child_samples | [1,3,5,10,15] | 15 |
| subsample | [0.6,0.7,0.8,0.9,1,1.1] | 0.01 |
| l2_leaf_reg | [1,5,10,15,20,25,30 | 0.7 |
| max_depth | [2,3,4,6,7,10] | 10 |

Table S3. Random Forest model

| Hyper-parameters | Value ranges | Optimal value |
| --- | --- | --- |
| n_estimators | [200,400,1000,1500] | 1000 |
| min_samples_split | [5,6,7,8,9,10,15] | 7 |
| max_features | [auto,sqrt,log2] | sqrt |
| max_depth | [4,5,6,7,8,9,10,15] | 15 |
